# BP180/Collagen XVII: A Molecular View

**DOI:** 10.3390/ijms222212233

**Published:** 2021-11-12

**Authors:** Jussi Tuusa, Nina Kokkonen, Kaisa Tasanen

**Affiliations:** PEDEGO Research Unit, Department of Dermatology, Medical Research Center Oulu, Oulu University Hospital and University of Oulu, P.O. Box 8000, FI-90014 Oulu, Finland; jussi.tuusa@oulu.fi (J.T.); nina.kokkonen@oulu.fi (N.K.)

**Keywords:** autoimmune disease, bullous pemphigoid, hemidesmosome, intrinsically disordered, keratinocyte, protein–protein interaction, transmembrane collagen

## Abstract

BP180 is a type II collagenous transmembrane protein and is best known as the major autoantigen in the blistering skin disease bullous pemphigoid (BP). The BP180 trimer is a central component in type I hemidesmosomes (HD), which cause the adhesion between epidermal keratinocytes and the basal lamina, but BP180 is also expressed in several non-HD locations, where its functions are poorly characterized. The immunological roles of intact and proteolytically processed BP180, relevant in BP, have been subject to intensive research, but novel functions in cell proliferation, differentiation, and aging have also recently been described. To better understand the multiple physiological functions of BP180, the focus should return to the protein itself. Here, we comprehensively review the properties of the BP180 molecule, present new data on the biochemical features of its intracellular domain, and discuss their significance with regard to BP180 folding and protein–protein interactions.

## 1. Introduction

BP180, also known as collagen XVII, was originally characterized as bullous pemphigoid (BP) autoantigen 2 (BPAG2), located in hemidesmosomes (HD) of epithelial cells [1,2]. BP is a blistering autoimmune skin disease. It is characterized by tense bullae, erythema, urticarial or eczematous lesions, and intense pruritus [3,4]. BP normally first manifests during old age (>75 years) [4,5]. BP is associated with several co-morbidities, including dermatitis herpetiformis and neurological diseases such as multiple sclerosis, Alzheimer’s disease, and Parkinson’s disease [6,7]. Furthermore, several drugs predispose the recipient to BP, including the dipeptidyl peptidase 4 inhibitors, which are used in the treatment of type 2 diabetes [8]. However, it is not known how these and other factors induce the loss of immunological tolerance toward self-antigens in BP. This is the decisive step that leads to the appearance of autoantibodies against the dermal-epidermal junction, the induction of inflammation, the recruitment of neutrophils, eosinophils, and mast cells, and finally to the loss of adhesion between the epidermal keratinocytes and the underlying basement membrane [9]. BP180 is the main autoantigen in BP targeted by the IgG, IgA, and IgE autoantibodies [9,10].

In addition to pemphigoid diseases, BP180 is also associated with genetic blistering skin diseases: missense or nonsense mutations in the *COL17A1* gene are found in patients with one subtype of junctional epidermolysis bullosa (JEB) (Table 1). The lack of BP180 and/or its disturbed function causes keratinocytes to detach from the basal lamina, which leads to the separation of the epidermis from the dermis, which results in blistering of the skin. The clinical features and detailed pathomechanisms of these diseases are described in detail elsewhere [3,4,11,12]. The intensive research of recent decades has hugely broadened the understanding of the role of BP180 in these pathological stages. However, the focus has been more on the features of genetic mutations, proteolytic cleavage patterns, and antigens, with less attention paid to the biochemistry of the wild type homeostatic BP180 protein itself.

**Figure 1:**
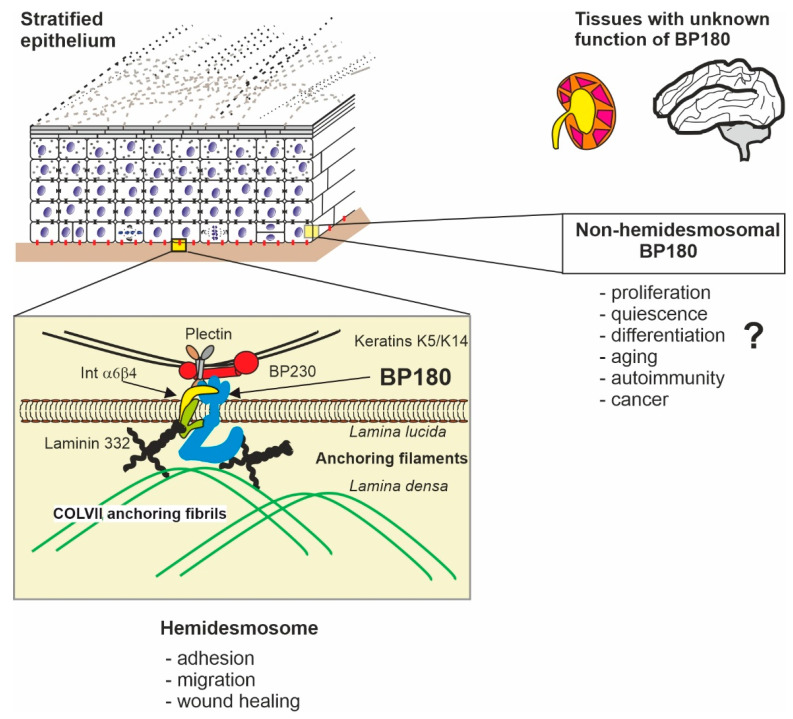
The locations and functions of BP180. The structure, function, and interactions of BP180 (cyan) are best characterized in cutaneous hemidesmosomes (HD, inset box) which create the adhesion between basal keratinocytes (the bottom layer of nuclear cells and the basal lamina (brown line). In HD, BP180 exists as a trimer, with the intracellular domain (ICD) binding to keratin intermediate filaments via plectin and BP230. The extracellular domain (ECD, “ectodomain”) of BP180 binds to integrin alpha 6, laminin-332, and collagen IV, which connect HD to collagen VII anchoring fibrils of the basal lamina. The non-HD functions of BP180 are relatively unknown, as are the functions in non-epithelial tissues such as the kidney and the brain. The sizes of macromolecules are not in scale and their molecular shapes are schematic. The mutual positions describe only roughly the sites of interaction.

The cDNAs that encode human and murine BP180 were published in the early 1990s [13,14]. The topology of the protein, including its cytosolic amino terminus, membrane spanning domain, and extracellular carboxy terminal domain containing discontinuous collagenous regions, was deduced from the sequence and confirmed using domain-specific antibodies [15,16]. BP180 is a type II transmembrane protein that belongs to the superfamily of transmembrane collagens. It has an apparently globular intracellular domain (ICD) and a large extracellular domain (ECD or “ectodomain”) that features collagenous domains interspersed with non-collagenous (NC) ones [13,17]. In the homotrimeric configuration of BP180, the ectodomains form a coiled-coil collagen triple helix, which undergoes a constitutive or induced shedding [18]. BP180 is expressed in several tissues, but its best understood function is that in the HDs of skin keratinocytes and other cells of the stratified epithelium: in these cells, BP180 contributes to the connections between the intracellular keratin filament network and extracellular proteins of the basement membrane, thus establishing adhesion between the epithelium and underlying basal lamina [16,19] (Figure 1).

Here we provide a comprehensive review of the BP180 protein, its structure, and its functions, both in the adhesion of epithelial cells and in emerging novel roles.

## 2. Structure of BP180

**Figure 2:**
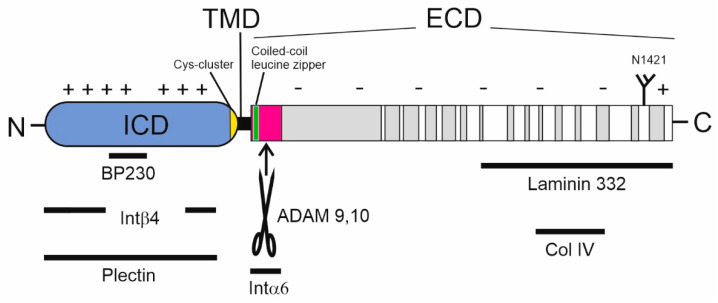
The primary structure of BP180 and its protein–protein interaction sites. The intracellular domain (ICD, blue) has a positive net charge and interacts with anionic regions of cytosolic/transmembrane proteins. The cytosolic cysteine cluster (yellow) of BP180 locates to the immediate vicinity of the transmembrane domain (TMD, black). The non-collagenous (NC) domain 16A (pink) contains a leucine zipper (green) and cleavage site(s) used in A disintegrin and metalloprotease (ADAM)—mediated shedding of the extracellular domain (ECM) of BP180. The other NC domains are shown in white and collagenous domains are denoted by gray shading. The charge distribution is indicated by ‘+’ and ‘−’. The bars show the most important protein–protein interaction sites and corresponding proteins, mapped mainly by yeast two-hybrid screens (see the main text). BP = bullous pemphigoid, Col = collagen, Int = integrin.

To date, sequence analyses have revealed several noteworthy features. Human BP180 (1497 aa) contains 15 collagenous domains that are flanked and interrupted by NC sequences in its extracellular domain (ECD, aa 489–497) (Figure 2) [13]. The collagenous domains harbor the repeating triplet glycine-X-Y, where X is often proline and Y hydroxyproline [20]. Mouse BP180 has a similar structure, with either 13 or 14 collagenous domains in each of its two transcriptional variants [14,21]. The interspersed NC domains are denominated from the carboxy terminus as NC1, NC2 up to NC16 (in human BP180). The NC domain closest to the amino terminus (human NC16, mouse NC14) consists of an extracellular juxtamembrane region, (NC16A; human aa 489–566), a transmembrane domain (NC16B; human aa 468–488), and an intracellular domain (ICD, NC16C; human aa-1–467) (Figure 2; https://www.uniprot.org/uniprot/Q9UMD9, accessed on 11 November 2021). Tryptophan 467 is sometimes considered as a part of the transmembrane domain [13], although as far as we know, its embedment into the lipid bilayer has not been investigated experimentally. The ICD contains four tandem repeats (aa 227–324), a glycine tract (aa 428–447), and a cysteine cluster (Cys456, 458, 461, and 462), the numbering of which refers to the human sequence. An early work showed that the ICDs of BP180 can form disulfide bridged trimers [22], but further research has not elucidated whether these exist in vivo and the reducing cytosolic environment makes this unlikely [23]. Interestingly, BP180 is a very charge-polarized protein. Its ICD is very basic (human ICD pI 9.5), whereas its ectodomain (aside from the very distal arginine-rich carboxy terminus) is acidic (pI 6.3). Without NC1, the human ectodomain has a pI of 5.7. Interestingly, the 36 amino acid-long NC4 domain (aa 1280–1315) of human BP180 contains mostly small polar amino acids (17 serines, 2 threonines, and 4 glycines). This property may play a role in protein–protein interactions and the NC4 domain may harbor several potential *O*-glycosylation sites (see below). Furthermore, this domain is located in a region that frequently acts as an autoantigen in BP [24].

The higher architecture of BP180 is a trimer, driven by the assembly of collagen triple helix [17,18,25,26,27,28]. Unlike fibrillar collagens, BP180 lacks a carboxy terminal propeptide. Instead, its collagen triple helix is induced by a coiled-coil structure between the leucine zipper-like sequences of the NC16A domain [18]. This kind of N-terminal triple helix seeding is typical of several other members of the transmembrane collagen family, such as collagens XIII, XXIII, and XXV [29,30,31]. Immunoelectron microscopy suggests that in the basement membrane the ectodomain extends through the lamina lucida into the lamina densa and then bends back into the lamina lucida [32,33] (Figure 1). Rotatory shadowed electron microscopy imaging of Triton X-100 solubilized purified BP180 has also demonstrated that its ectodomain has a rod-like morphology, while the ICD appears to be globular [17,34].

**Figure 3:**
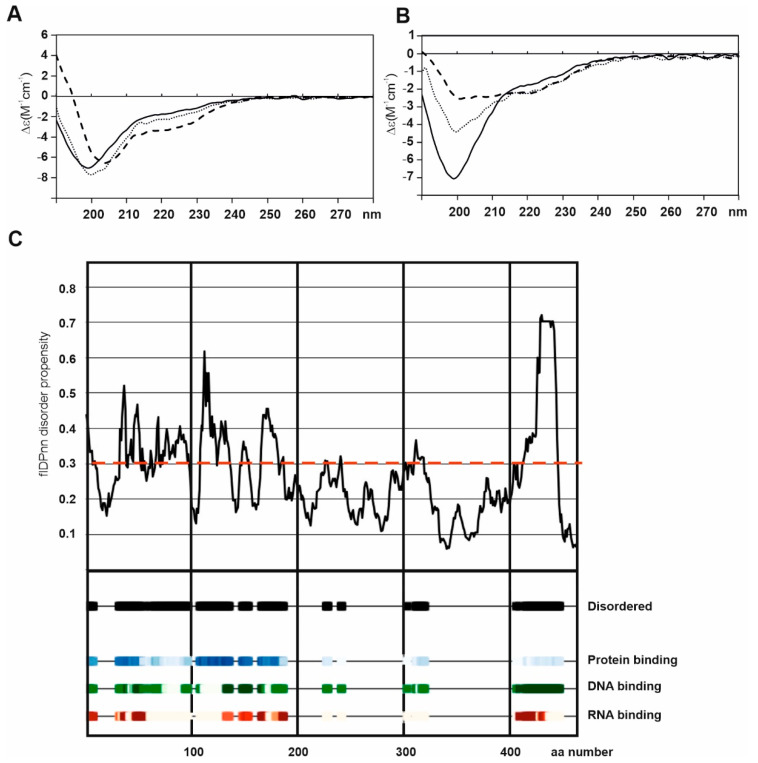
The intrinsically disordered structure of BP180 ICD can undergo charge-induced folding. The intracellular domain (ICD) of BP180 expressed in *Escherichia coli* was analyzed by circular dichroism spectroscopy. The continuous curve in (**A**,**B**) shows the spectra of purified soluble BP180 ICD to be typical of that of a random coil with the minimum at 195–200 nm. The addition of negatively charged detergent sodium dodecyl sulfate (**A**) or anionic dimyristoylphosphatidylcholine-dimyristoyliphosphatidylglycerol (DMPC-DMPG) (1:1) lipid vesicles (**B**) induces partial folding as observed by increased Δε below 200 nm, a shift of the minimum toward longer wavelength, and alpha helix-like bending of the curve (dashed curves). The dotted curve in (**A**) represents the spectrum of the BP180 ICD with neutral lipid n-dodecyl phosphocholine and in (**B**) with DMPC-DMPG vesicles + 1mM CaCl_2_ (reproduced from Tuusa et al. [36], according to Creative Commons Attribution 4.0 International License (http://creativecommons.org/licenses/by/4.0/, accessed on 11 November 2021). (**C**) New modeling was done for ICD of human BP180 by flDPnn program to map the disordered and protein/nucleic acids binding regions. Regions with values greater than 0.3 are considered to be disordered.

Although the ectodomain of BP180 shares features with the other transmembrane collagens, its ICD differs in that its intracellular N-terminus has a molecular weight of 50 kDa, in contrast to the short stub characteristic of other family members [35,36]. We have recently reported that the bacterially expressed ICD of human BP180 is intrinsically (~70%) disordered [36]. Our sequence analysis predicted a few secondary structure elements in the N-terminal half of the ICD and a larger folded domain between aa 235–425. This hypothesis was supported by the instability/stability of corresponding glutathione-S-transferase tagged fragments expressed in *E. coli*. Importantly, circular dichroism spectroscopy of the ICD (aa 2–455) showed spectra typical of a random coil, suggesting that the ICD of BP180 is intrinsically disordered. Even though the lack of eukaryotic chaperones and post-translational modification may explain the absence of a global fold, the ICD underwent a partial folding when anionic membrane lipid mimics were added (Figure 3a,b) [36]. This disordered nature and potency to fold may demonstrate that interacting macromolecules such as anionic membrane lipids and acidic interacting proteins may be required for the folding of BP180, suggesting that the ICD might be relatively unstable even as a part of the HD complex. This hypothesis is supported by a new model incorporating a recently published flDPnn algorithm [37] which predicts protein and/or nucleic acid interactions with disordered regions of the ICD of BP180 (Figure 3c). It seems that the under-investigated field of intrinsic disorder in keratinocyte proteomes is gathering pace [38]. The implications of these intrinsic structural features of BP180 to its function as a component of HD and other structures are discussed in Section 4, Section 6, and Section 7 below.

## 3. Expression of BP180

Human BP180 is expressed most abundantly in skin epithelial cells [39] and other stratified epithelia such as those of the oral mucosa [40], cornea [41], cervix [42], and vagina [43]. It is also abundant in the squamous epithelia and in the syncytial and cytotrophoplastic cells of the placenta [44,45]. The expression of BP180 by ameloblasts in the oral cavity is necessary for the formation of tooth enamel [46]. BP180 is also detected at low levels in the simple epithelia of the mammary glands [39] and intestines [47] although those tissues have type II HDs which lack BP180 and BP230 [48]. Altered, often high, expression of BP180 is found in many epithelial malignancies [44,49,50] including skin cancers [51,52], oral mucosa cancer [40,53], and colorectal cancer [47]. The role of BP180 in cancer was recently extensively reviewed by Jones and co-workers [54]. In layered epithelia, BP180 is predominantly expressed in basal cells and most notably in type I HDs [34]. In the skin, it is expressed in both the hair follicles and the interfollicular epidermis [55,56]. In addition to hemidesmosomal expression, non-hemidesmosomal BP180 is also present in the apicolateral surfaces of Triton X-100 soluble membranes of basal keratinocytes, suggesting a non-lipid raft localization [56,57]. While the hemidesmosomal expression and function of BP180 have been well described, its non-hemidesmosomal expression and—possibly adherent junction related—functions remain poorly characterized.

Credible evidence also demonstrates local BP180 expression in the kidney [58], in rodent and bovine retina [59], and in parts of the brain [60]. However, data from the Bgee and Expression Atlas databases show that if global expression of BP180 is detectable at all in these anatomical locations, expression levels are very low, being comparable to those seen in blood vessels and muscle and nerve tissue [61,62]. An interesting open question concerns the extent, location, and timing of neural expression of BP180 in the brain [59,60,63,64,65,66]. The RNA sequencing data in the Expression Atlas [62] show a low level of expression in the striatum, limbic system, and cortex. This corroborates the original finding of Seppänen and co-workers that BP180 is expressed in the pyramidal neurons of the deepest cortical layer as well as in the hippocampus and amygdala [60]. The function of BP180 in the nervous system is, however, completely unknown. BP180 is present in the kidney glomerular basement membrane and in the podocyte foot processes, making the kidney glomerulus another exciting, but under-investigated anatomic site [58]. BP180 knockout (*Col17a1* −/−) mice have smaller glomeruli than those of wild type, which show morphological abnormalities in the foot processes and glomerular basement membrane, albeit with no apparent deficit in kidney function. Importantly, most of the null homozygous mice die within a few weeks of birth, which may hide any actual functional consequences of their glomerular abnormalities [58,67].

Recently, an interesting new aspect of extracutaneous function of BP180 was pinpointed in the bone marrow mesenchymal stem cells, where BP180 regulates granulopoiesis [68]. BP180 restricts the production of granulocyte colony stimulating factor (G-CSF) and NF-κB expression, thereby regulating the biogenesis and liberation of granulocytes. Regulation is disrupted in mice whose NC14A domain has been deleted [68]. Further research on the function of BP180 in these novel roles and locations is eagerly awaited.

## 4. Post-Translational Modifications of BP180

The ectodomain of BP180 does not contain any cysteines, a feature that prevents the existence of intra- or interchain disulfides and of sulfur bridges to other proteins. In contrast, the existence of the proposed cytoplasmic disulfides in vivo, as discussed above [22], remains to be confirmed, as does the possible presence of palmitoylation, which has been observed in 17ODYA labeling [69].

**Figure 4:**
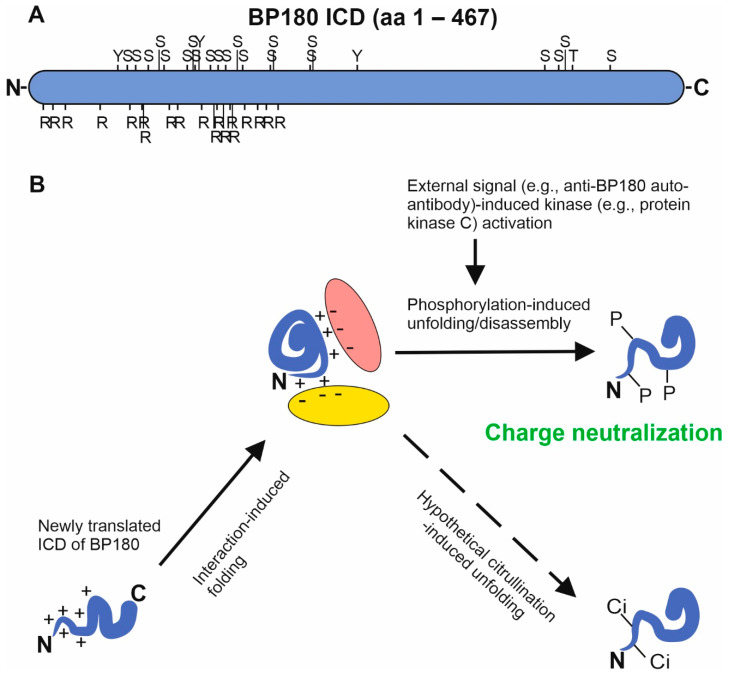
Charge changing post-translational modifications of BP180 ICD. (**A**) The localizations of serine (S), threonine (T), and tyrosine (Y) residues which are putative phosphorylation sites with >0.9 score in NetPhos 3.1 analysis and arginines (R) present in BP180 ICD. (**B**) A speculative model, how protein–protein interactions with negative charged proteins (represented by yellow and orange ellipses) may drive folding of BP180 ICD (blue), and the phosphorylation and/or hypothetical citrullination may lead to protein complex disassembly and BP180 ICD unfolding. P = phosphate group. Ci = citrulline. Aa = amino acid.

The ICD of human BP180 contains numerous putative phosphorylation sites: 75 serine, 57 threonine, and 11 tyrosine residues. A NetPhos 3.1 analysis [70] identified (J. Tuusa unpublished data) 59 serines, 40 threonines, and 5 tyrosines in the ICD as possible phosphorylation sites with scores > 0.5, a finding augmented by the presence of consensus sequences of protein kinase C (PKC) and/or cyclin-dependent kinase (Cdk) acceptor sites. With a stricter cut-off score (0.9) the NetPhos 3.1 server predicted phosphorylation at 21 serine residues, a single threonine, and three tyrosine residues, which are clustered in the N-terminus: 17 of 25 high score acceptor sites are located between aa 64 and 174 (J. Tuusa, unpublished data, Figure 4). Experiments with PKC activators and inhibitors suggest that calcium-induced differentiation of keratinocytes occurs simultaneously with PKC-mediated phosphorylation of the ICD of BP180 at serine residues and HD disassembly [71,72]. The most potent phosphorylation sites are located in the N-terminal half of the ICD, which without phosphate is very basic due to high number of lysine and arginine residues. While lysine residues are relatively regularly distributed, arginines have a highly polarized pattern of distribution: the region between aa 1 and 177 of human BP180 contains 19 arginines while the sequence aa 178–467 harbors none (Figure 4). Based on the current data, it can be hypothesized that charge neutralization by phosphorylation, resulting in loss of electrostatic interactions and the unfolding of ICD plays a role at least in HD disassembly and may also be involved in the autoantibody induced BP180 internalization in BP (Figure 4) [73].

In addition to multiple putative intracellular sites, BP180 has an interesting rare extracellular phosphorylation site at a serine residue. Serine 544 of the human BP180 NC16A domain is phosphorylated by casein kinase II, which creates an enhanced epitope for BP autoantibodies and may have a regulatory physiological role since it inhibits ectodomain shedding in vivo [24,74,75].

Citrullination, which is an irreversible, charge-neutralizing deamidation reaction at arginine residues, is a common modification implicated in autoimmune and other diseases associated with collagens and other ECM components [76,77,78]. The ectodomain of human BP180 contains 49 arginines, including a cluster of five residues (located at aa 1487–1491). While hypothetical deamidation of the ICD might have effects on folding/unfolding or protein–protein interactions, and overall citrullination might create autoantigenic epitopes, to the best of our knowledge, citrullination has not been reported in either the ICD or the ectodomain of BP180.

Polyubiquitination has been reported for BP180 targeted to proteasomal degradation [79], but other isopeptide modification like SUMOylation have not been described. Moreover, aside from putative palmitoylation [69], no lipid modifications have been reported.

Finally, the ectodomain harbors at least four additional and only partly investigated posttranslational modifications, *N*-/*O*-glycosylation and proline/lysine hydroxylation [22,27,80]. The surface expression of mature BP180 requires the *N*-glycosylation of asparagine 1421 since BP180 with glycosylation preventing mutation Asn1421Thr accumulates in perinuclear endoplasmic reticulum [81]. Proline and lysine hydroxylation takes place with a hydroxyproline/proline ratio of 0.4 and a hydroxylysine/lysine ratio of 0.5; these modifications are necessary for complete collagen triple helix formation [27,80]. A minor proportion (3.3%) of BP180 ectodomain hydroxylysines become glycosylated [27]. The presence of mucin type *O*-glycans is possible: at least Ser811, Ser905, Ser1293, Ser1298, Ser1306, Ser1309, and Ser1312, all located in the non-collagenous domains, are predicted by the NetOGlyc 4.0 server [82] to be putative *O*-glycan acceptor sites with scores of >0.9 (J. Tuusa unpublished data). However, this has not been empirically verified.

## 5. BP180 Ectodomain Shedding

The autoantibodies in BP patient sera recognize two forms of BP180: 180 kDa and 120 kDa, both of which are present in the skin [83]. Keratinocyte cell extract contains both forms whereas only the 120 kDa form is found in cell culture medium [84]. The 120 kDa form is the soluble BP180 ectodomain, which is proteolytically shed from the full-length BP180 constitutively and in an inducible manner by A disintegrin and metalloprotease (ADAM) 9 and 10 [18,83,84,85,86,87]. ADAM17 as well as furin can also induce shedding and their inhibition prevents the cleavage of BP180, but the requirement of these proteases may be conditional and/or indirect [84,86]. In cell culture, BP180’s sites of cleavage by ADAMs have been mapped by mass spectrometric analysis to Gly513|Asp514, Arg523|Leu524, Leu524|Gln525, and Gln525|Gly526 [88]. While Leu524|Gln525 is the dominant physiological constitutive cleavage site, the shedding is shifted to Arg523|Leu524 in migrating keratinocytes [87]. Despite the existence of dominant cleavage sites, ADAMs are not sequence specific, but rather cleave on a conformational basis at a constant distance from the plasma membrane. Indeed, large deletions comprising the whole NC16A domain (aa 490–566) or a major part of it (aa 504–558) prevent the shedding of human BP180, while mutants with smaller deletions covering the areas of aa 504–520, 521–540, or 540–566 do undergo cleavage [84]. However, a mutant BP180 with aa 528–547 deleted is not cleaved in vitro [84]. This implies that shedding is independent of the exact sequence but rather determined by structural features. This in vitro finding is likely applicable to the in vivo process. The mouse protein with a genetic ablation of aa 498–569 of the NC14A domain (which corresponds to human NC16A) is still shed [89]. However, in another mouse model deletion of aa 513–544 led to a non-shedding phenotype [90]. The coiled-coil sequences were spared in the latter model, suggesting that structural differences between mutant ectodomains may explain the distinct susceptibilities to shedding. While furin does not act as a physiological sheddase, it activates ADAMs and cleaves monomeric BP180 at a specific Arg-Ile-Arg-Arg sequence (aa 504–507), which is in the coiled-coil region and is exposed only if the normal trimeric configuration is distorted [18].

The shedding of BP180 is dependent on the membrane microenvironment [91]. Native HDs do not form in cultured human keratinocytes and fibroblasts; BP180 is present in HD-like structures and co-localizes with lipid raft markers. Furthermore, depletion of cholesterol enhances shedding [91], probably by liberating BP180 from a lipid-ordered phase to become an ADAM substrate. These findings may be especially relevant for the apicolateral non-hemidesmosomal BP180.

The shed ectodomain of 120 kDa may undergo further processing. Patients with linear IgA bullous dermatosis, a pemphigoid group disease (Table 1), have IgA autoantibodies that recognize the 120 kDa ectodomain and the plasmin generated 97 kDa fragment with a higher affinity than they do the full length 180 kDa protein [92,93,94]. Plasmin cuts the 120 kDa ectodomain from the carboxy terminus, processes the amino terminus, and generates a 97 kDa fragment in vitro in the presence of ADAM inhibitors [92,93,94]. In addition to the novel termini, this processing creates neoepitopes which map to the 15th collagenous domain via an unknown mechanism, but the appearance of novel conformational epitopes has been proposed [94].

## 6. Protein–Protein Interactions of BP180

In the skin, the protein–protein interactions between BP180 and the other HD components are well understood (Figure 1 and Figure 2), but the binding partners of BP180 in other tissues are largely unknown. The overall structure of the HD is described in several reviews [48,95,96]. HDs anchor the cytoskeletal intermediate filament bundles to the basement membrane. An individual keratin filament is bound by the two HD associated plakin family proteins, plectin and BP230 (also called BPAG1). Superresolution microscopy has revealed that the core HD transmembrane proteins integrin α6β4 heterodimers and BP180 trimers are located around the keratin–plectin/BP230 contact points [97]. Importantly, the ICD of BP180 is needed for proper linking of integrin β4 to BP230 [98]. The protein–protein interactions of the BP180 ICD with keratin K18, plectin, BP230, and the cytosolic domain of integrin β4 have been mapped using yeast two-hybrid screening [39,99,100,101]. The BP180 regions essential for the keratin K18 and BP230 interactions (aa 13–89 and 145–230) have pIs of 10.3 and 10.0, respectively, while the interacting part of keratin K18 (aa 332–429) has a pI of 4.8 and BP230 (aa 1–555) a pI of 5.4. Similarly, the fibronectin domain III of integrin β4 (aa 1530–1625), which interacts with the positively charged ICD of BP180, is slightly acidic with pI 6.2, and the interacting region of Plectin (aa 563–819) is near neutral (pI 6,7) [36,100]. Furthermore, the acidic regulatory protein 14-3-3σ binds to the ICD of BP180 [102]. Thus, it is likely that electrostatic interaction with anionic proteins and the negatively charged cytosolic leaflet of the plasma membrane regulates the folding and unfolding of ICD, thus contributing to the type I HD assembly/disassembly. It can be speculated that a process of charge neutralization by phosphorylation of the positively charged ICD of BP180 may drive the HD disassembly by the unfolding of ICD. Non-hemidesmosomal proteins such as actinins 1 and 4 and p120 catenin, implicated in adherent junctions, can also bind to the ICD of BP180 and interestingly, they also harbor a negative net charge [103,104].

The BP180 ectodomain binds directly to integrin α6, laminin-332, and collagen IV, and these interactions mediate the adhesion of keratinocytes to the matrix of the basal lamina and, together with ectodomain shedding, regulate keratinocyte migration [98,105,106,107]. In cases of BP, these interactions are disrupted by autoantibodies against BP180 [108,109,110]. In addition to *COL17A1* mutations leading to truncated translation products and almost complete loss of BP180 polypeptide, mutations that affect the protein–protein interactions between BP180 and components of the basement membrane have been detected in patients with JEB [111]. For example, the presence of the Arg1303Gln mutation in the serine-rich NC4 domain, does not affect protein stability, but demolishes the binding of BP180 to laminin-332 [112,113]. These examples demonstrate how genetic mutations translate into defects in hemidesmosomal adhesion. Similarly, strong in vitro evidence shows that autoantibodies blocking BP180-collagen IV interaction can demolish cell matrix interactions [110].

In addition, there are interactome reports of the binding or proximity of BP180 with periplakin, DFNB31, CSTF2T, PPLIL1, PLOD3, CDH1, PNKP, HRAS, SPDL1, EGFR, and ubiquilin2, which may be involved in the biosynthesis, transport, and post-translational modification of BP180 or its non-hemidesmosomal functions. These interactions are compiled in the BioGRID interaction database [114].

## 7. The Role of BP180 in Cell Migration, Proliferation, and Differentiation

As previously stated, HDs are needed to keep keratinocytes fixed to the underlying basal lamina. Furthermore, remodeling of HDs is essential for cell migration during growth and wound healing. This requires both the creation of novel adhesion between the extracellular matrix and lamellipodial cell extension on the ‘front’ side (the side aligned with the direction of migration) and the loss of adhesion at the ‘rear’ side of the cell [48,115,116]. In wound healing, the migration and proliferation of basal cells are both required and both are affected by the proteolytic cleavage of BP180 [106,117,118].

Keratinocyte migration along the basal lamina is regulated by the shedding of the BP180 ectodomain by ADAM metalloproteases [85]. The shed ectodomain is deposited into the matrix of the basement membrane and can be extracted from the epidermis [119]. In vitro migrating keratinocytes also leave behind tracks consisting of laminin-332 bound to BP180 ectodomain [109]. During wound healing, the expression and shedding of BP180 are strongly induced in basal keratinocytes [40,50,90,106]. At least in mice, BP180 shedding decelerates wound closure [106], which supports the finding of in vitro experiments that have shown that keratinocyte migration is suppressed by exogenous addition of soluble ectodomain [85]. Shedding may also play a role in BP180 turnover, HD disassembly and the balance between proliferation and differentiation of epithelial cells, but the data are scarce at the moment.

It is not known if membrane-tethered BP180 endodomain (ICD + transmembrane domain) generated by shedding has intrinsic signaling functions. The endodomain alone can rescue lost lamellipodial polarity in cells that lack BP180, but this may be due to structural stabilization [98,106]. Ectodomain shedding suppresses the proliferation and migration of keratinocytes by dampening integrin α6β4-PI3K-Akt-mTOR signaling [106]. Since the recombinant ectodomain does not inhibit this pathway, it is possible that the shedding event or the shed endodomain itself mediates the suppressive signal or that full-length BP180 is required for activation [106]. The inhibition of Akt-mTOR signaling by BP180 also suppresses the proliferation of breast cancer cells in 2D and 3D cell culture models [120]. While BP180 seems to be upstream from TGFβ signaling in hair follicle stem cells [121] and Wnt signaling in interfollicular stem cells [56], the specific roles of the BP180 ectodomain and ICD are unknown. Galiger and co-workers hypothesized that the rescue of proliferation by BP180 endodomain expression follows direct interaction between the endodomain and cell cycle regulating signaling pathway [117]. This remains to be experimentally verified.

It appears that BP180 may also have a more fundamental role in the regulation of cell proliferation and differentiation. It has been shown that in nestin-positive hair follicle-associated pluripotent (HAP) stem cells, the expression of BP180 is high, decreases during colony formation, and increases again when the cells differentiate into basal keratinocytes [122]. In both hair follicles and in the interfollicular epidermis, the loss of BP180 leads to aging by reducing the renewal of stem cells by symmetric cell division [55,123].

As well as being potential keratinocytes, HAP cells can also differentiate into one of several other cell types, including neurons, glial cells, and smooth muscle cells [122]. The expression of BP180 in these cell types is low or absent, and the interesting question is whether BP180 plays a decisive role in determining the fate of HAP cells [122]. The shed 120 kDa ectodomain stabilizes laminin-332, and shedding by ADAMs is required for epithelial-mesenchymal transition in lung cancer spheroid cultures via the FAK/AKT/GSK3β signaling pathway [124]. The same BP180-induced pathway also maintains the features of cancer stem cells by up-regulating glycolysis and oxidative phosphorylation [125]. On the other hand, an increased expression of BP180 can suppress reactive oxygen species-mediated apoptosis in multilayered transformed epithelia [126].

## 8. Mechanisms and Consequences of BP180 Downregulation

The molecular mechanisms leading to BP180 degradation as well as the downstream signaling pathways, which are regulated by the loss of BP180, are still largely to be discovered. Downregulation of BP180 during normal development and physiology differs from pathological states, and the degradation of hemidesmosomal and non-hemidesmosomal BP180 are likely different processes that may involve both extracellular proteases and intracellular degradation after endocytosis.

During HD disassembly, the calcium dependent phosphorylation of BP180 precedes its downregulation in a process wherein several other HD components, such as integrin β4 and plectin, undergo phosphorylation or dephosphorylation involving PKC, protein kinase D (PKD), extracellular signal-regulated kinase (ERK), and calcineurin phosphatase [71,127,128,129]. BP autoantibodies and monoclonal antibodies against the immunodominant NC16A domain of BP180 induce macropinocytosis-mediated internalization of BP180 [73]. This process is dependent on the phosphorylation of the BP180 ICD by PKC [130]. Ujiie and co-workers have shown that IgG-dependent but complement-independent downregulation of BP180 is mediated by polyubiquitylation and proteasomal degradation [79]. Proteins that enter the cell via macropinocytosis are usually degraded in lysosomes. However, proteasomal degradation has been reported in the context of antigen cross-presentation on the MHC-I pathway [131]. Currently, it is not known whether BP180, which becomes internalized by macropinocytosis, is degraded solely by the proteasomal pathway, or if lysosomes or other routes of endocytosis may also be involved.

The role played by ADAM-mediated shedding in the downregulation of BP180 during normal physiological turnover remains unclear. In pathological conditions, e.g., in linear IgA dermatosis, plasmin can cleave BP180 [93] and in BP mast cell and/or basophil-derived Granzyme B can directly degrade BP180 [132]. Neutrophil elastase has been shown to degrade hemidesmosomal BP180 in a murine model of BP [133]. It also degrades BP180 in hair follicle stem cells in response to DNA damage and during aging [55]. The role of proteases in the loss of apicolateral non-hemidesmosomal BP180 in the interfollicular epidermis is unclear, while in cultivated keratinocytes, UV-induced degradation of BP180 is catalyzed by marimastat-sensitive proteases, likely matrix metalloproteases (MMPs) or ADAMs [123]. In squamous cell cancer, MMP-9 catalyzes the cleavage of BP180, which results in the shedding of the BP180 ectodomain [134]. It remains to be seen whether these protease activities found in pathological conditions also regulate the degradation of BP180 during the differentiation and migration of keratinocytes and cutaneous aging.

In hair follicle stem cells, the loss of BP180 leads to attenuated TGFβ-Smad signaling, which downregulates the maintenance of the neighboring melanocyte stem cells in paracrine fashion, in contrast to the autocrine regulation seen in basal keratinocytes [56,121]. In interfollicular keratinocytes, the genetic ablation of BP180 does not directly inhibit the mitotic cell cycle, but leads to loss of quiescence and to transient hyperproliferation, which eventually result in aging. This is mediated by the Wnt-β-catenin pathway, which is itself dependent on BP180 expression. In wild-type mice, normal aging is accompanied by a reduction of BP180 levels in basal keratinocytes. Notably, BP180 is lost from the apicolateral non-hemidesmosomal membranes, while hemidesmosomes still contain BP180. This change is regulated by the calcium-dependent atypical protein kinase C (aPKC) [56], which is a major polarity inducer and interacts with BP180 via Partition-defective(PAR)3 protein [135]. The substrates of aPKC in this process are unknown, but it is intriguing to speculate that the ICD of BP180 might be one.

**Table 1:** Diseases with BP180 involvement.

	**Molecular Defects**	**References [3,11,45,47,52,53,110,111,124,136,137,138,139,140,141,142]**
**Hereditary blistering skin diseases** Intermediate junctional epidermolysis bullosa (JEB)	Recessive missense and nonsense mutation in several sites in *COL17A1*	Bauer and Lanschuetzer, 2003 Kiritsi et al., 2011 Huber et al., 2002 Has et al., 2019
**Autoimmune skin diseases** Bullous pemphigoid Mucous membrane pemphigoid Gestational pemphigoid Linear IgA bullous dermatosis	IgG, IgE, IgA autoantibodies against the NC16A immune-dominant domain of BP180 and other epitopes located in the ICD, Col15 domain, and C-terminus	Bağcı et al., 2017 Xu et al., 2013 Kamaguchi and Iwata, 2019 Solano-Lopez et al., 2015 Huilaja et al., 2014 Cozzani et al., 2020
**Cancer** Squamous cell carcinoma Basal cell carcinoma Ameloblastoma Colon cancer Lung cancer Melanoma	Altered expression of BP180 Altered shedding of BP180	Moilanen et al., 2015, 2017 Parikka et al., 2001 Liu et al., 2016a,b Krenacs et al., 2012

## 9. BP180 in Health and Diseases—Future Views

BP180 was originally identified as a hemidesmosomal protein acting as a major autoantigen in pemphigoid diseases. Subsequently, BP180 mutations have been implicated in JEB and altered expression in cancer (Table 1) illustrating the important biological and pathological functions of epidermal cells, especially keratinocytes.

What remains to be discovered? The function of non-hemidesmosomal BP180 in basal keratinocytes is still poorly understood, and almost nothing is known about its role in the brain, kidney, and placenta. We are only just beginning to unravel the signaling pathways that connect BP180 to the regulation of various physiological processes such as differentiation, migration, and aging of various cells and tissues. A deeper understanding is needed of the dynamics of the structure of BP180, including the intrinsically disordered character of its ICD in the absence of its interaction partners. The physiological and pathological processing and degradation of BP180 should be investigated more carefully, and this may help us to understand, for example, how immunological tolerance of BP180 is lost in the patients with BP.

## Data Availability

Bioinformatic data indicated as “unpublished” is available from authors.
